# Intraosseous line insertion for the primary health care physician

**DOI:** 10.4102/safp.v65i1.5691

**Published:** 2023-03-24

**Authors:** Indiran Govender, Henry I. Okonta, Olukayode Adeleke, Selvandran Rangiah

**Affiliations:** 1 Department of Family Medicine and Primary Health Care, Faculty of Health Sciences, Sefako Makgatho Health Sciences University, Pretoria, South Africa; 2 Department of Family Medicine and Rural Health, Faculty of Health Sciences, Walter Sisulu University, Mthatha, South Africa; 3 Department of Family Medicine, Faculty of Health Sciences, University of KwaZulu-Natal, Durban, South Africa

**Keywords:** intraosseous line, primary healthcare physician, children, resuscitation, intra vascular access

## Abstract

Early rapid access to the vascular system is essential in emergencies and is lifesaving. In this article, we will provide information on the common sites used, the equipment that is required, the indications and contraindications for intraosseous line insertion, how to correctly and safely do the procedure, medication that can be administered, post insertion line management and possible complications. This is a lifesaving procedure and primary healthcare physicians should acquire this skill.

## Introduction

Early, quick access to the circulation is vital in the critically ill or injured patients. The preferred route via large accessible veins is often not accessible and readily available in children who are dehydrated. Intraosseous (IO) infusion is a vascular access technique by which a specialised hollow-bore needle is placed through the cortex of a bone into the medullary sinuses.^1,2^ The primary health care physician is often the first responder who is required to initiate vascular access. Although the approach was first described in the 1920s by Dr Cecil K. Drinker, it was only in the 1980s that it was widely used worldwide.^3^

This procedure is used to access the systemic circulation through the non-collapsible venous plexuses of the bone marrow if peripheral intravenous access cannot be quickly or reliably established.^3^ It is safe and applicable to patients of all age groups.^4,5,6^ It can be achieved much faster than intravenous access, thus providing rapid, lifesaving intravascular access in challenging environments. Despite these features and advantages, this procedure is often not utilised in primary care because of a lack of awareness and training. This article will provide information on the essential elements of the intraosseous line insertion procedure for the primary care physician. In order to acquire this important skill, it is important to know the common sites used, the equipment that is required, the indications and contraindications for intraosseous line insertion, how to correctly and safely do the procedure, the medications that could be administered, post-insertion line management and the possible complications.

## Common intraosseous sites

The commonly used sites for intraosseous line placement include the manubrium, proximal humerus, iliac crest, distal femur, proximal tibia, distal tibia and calcaneus. The preferred sites in adults are the proximal tibia, proximal humerus and sternum while the distal femur, proximal tibia and distal tibia are the preferred sites in infants and newborns.^2,3^

## Indications

Intraosseous access is indicated in situations where immediate venous access is required such as cardiopulmonary arrest and hypovolemic shock. It is also indicated in instances of delay or difficulty to establish venous access such as sepsis, burns, obesity, oedema and seizures. Other indications include blood draws, medication infusion, local anaesthesia and contrast injection for radiologic evaluation.^4,7^

## Contraindications

The intraosseous route is contraindicated if there is adequate venous access. It is also contraindicated in the following conditions: infection at the insertion site, fracture of the bony site, ipsilateral fracture of the extremity, burn at the insertion site, previous intraosseous attempted site, previous attempt in a different location on the same bone, previous intraosseous site less than 48 h, inability to locate landmarks, osteogenesis imperfecta, osteoporosis and osteopetrosis.^8,9^ Caution should be taken in patients with right to left intracardiac shunts because of the risk of fat or bone marrow emboli.^9^

## Equipment for intraosseous access

**FIGURE 1:**
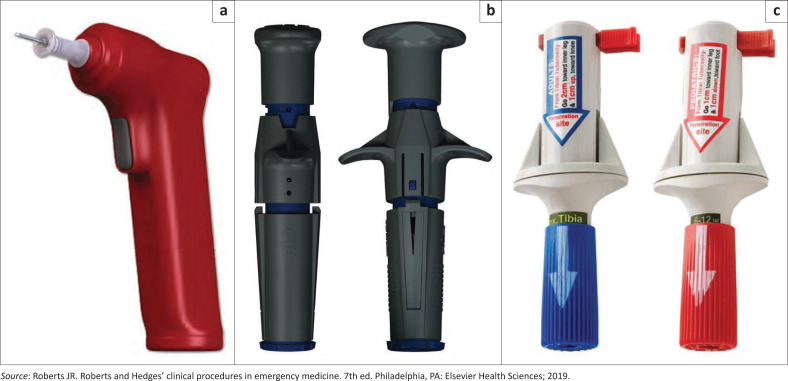
Mechanical intraosseous devices. (a) EZ-IO device, (b) NIO intraosseous device, (c) bone injection gun (BIG).

The equipment required for intraosseous line insertion includes the following: sterile gloves, antiseptic swab (alcohol swab or cotton wool ball soaked in antiseptic solution), fenestrated sterile drape, lignocaine 1%, 5 mL – 10 mL syringe, intraosseous needle device (refer to Figure 1^11^ for sites), spinal needle for neonates, sterile infusion set with intravenous (IV) fluid, adhesive strapping, elastic bandage and padded splint.^4,10^

**FIGURE 2:**
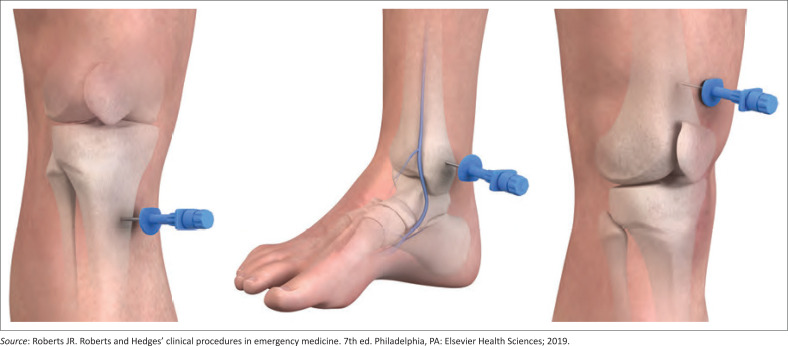
Some common intraosseous insertion sites.

**FIGURE 3:**
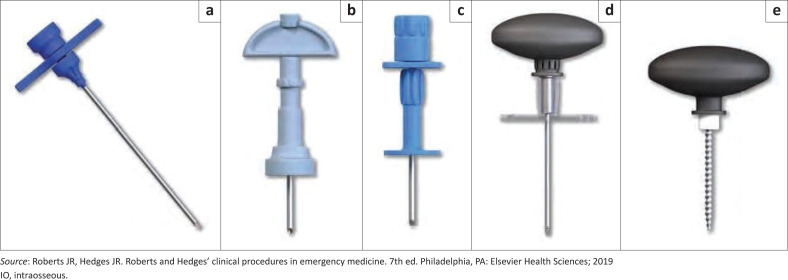
Manual interosseous needles. (a) Jamshidi bone marrow aspiraon needle, (b) Illinois sternal/Iliac aspiraon needle, (c) Jamshidi disposable sternal/Iliac aspiraon needle, (d) Cook IO needle, (e) Sur-fast needle.

There are several devices for intraosseous insertion. The mechanical devices include the BIG (bone injection gun), arrow EZ-IO^®^, FAST1^™^ intraosseous infusion system and NIO (New intraosseous) device (Figure 2).^11^ The Jamshidi needle, Cook needle, Sur-Fast intraosseous needle and the 22-gauge needle (used only for neonates) are manual devices. The procedure is much faster, with a much higher success rate at the first attempt when mechanical devices are used compared to manual devices refer to Figure 3^11^ for types of needles.^4,10,11^

## Procedure

**FIGURE 4:**
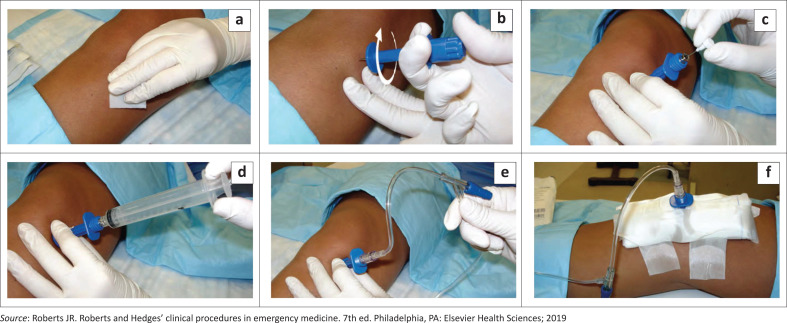
Procedure to insert the intra osseous line.

The procedure, risks and benefits are explained to the patient, parent or guardian if the patient is a minor and consent is taken. Use a standard sterile technique (see Figure 4a). Specifically, wash your hands and put on sterile gloves, then clean and disinfect the insertion site. (Refer to Table 1^4^ for the best device to be used for each site) The procedure is generally well tolerated without anaesthetics,^2,7^ but 1% lignocaine may be infiltrated into the insertion point in the conscious patients to reduce pain.^1,2,12^ Position the patient to ensure full access to the insertion site. Identify the landmarks and palpate both margins of the bony site to ensure penetration of the bone centrally. Refer to Table 2^4^ for bone insertion sites. Avoid insertion into the epiphyseal plate in children.

**TABLE 1:** Intra osseous insertion site and the device to be used.

Site	Adult	Children	Devices
Sternum	Yes	No	Manual, FAST1™
Humeral head	Yes	No	Manual, BIG, EZ-IO^®^
Distal radius	Yes	No	Manual
Distal ulna	Yes	No	Manual
Iliac crest	Yes	No	Manual
Distal femur	Yes	Yes	Manual, BIG, EZ-IO^®^
Proximal tibia	Yes	Yes	Manual, BIG, EZ-IO^®^
Distal tibia	Yes	Yes	Manual, BIG, EZ-IO^®^

*Source:* Tay ET, Rowe VL. Intraosseus access. Drugs Dis [serial online]. 2022. Available from: https://reference.medscape.com/article/80431

BIG, bone injection gun.

**TABLE 2:** Commonly used bone sites and location of their insertion points.

Bone sites	Point of insertion
Manubrium	1 cm below the sternal notch
Proximal Humerus	With the humerus held in internal rotation, elbow flexed to 90° and hand placed on the abdomen, the needle is placed 2 cm above the surgical neck into the greater tubercle at 45° to the anterior plane.
Distal femur	1 cm proximal to the patella and 1 cm to 2 cm medially
Proximal tibia	1 cm inferior and 1 cm medial to the tibial tuberosity
Distal tibia	2 cm proximal to the medial malleolus.

*Source*: Tay ET, Rowe VL. Intraosseus access. Drugs Dis [serial online]. 2022. Available from: https://reference.medscape.com/article/80431

Insert the intraosseous needle pressing through the skin and perpendicular to the surface, until the tip of the needle touches the bone. For manual intraosseous devices, apply steady manual pressure with a twisting or rotatory motion (but not a rocking motion) to drill the needle through the bone cortex (Figure 4b). With automatic intraosseous devices, squeeze the driver trigger and apply a minimal amount of gentle, steady pressure to drill the bone cortex.^13^ Stop drilling immediately when a sudden ‘give’ or ‘pop’ is felt as this indicates entry into the medullary cavity. With the needle correctly inserted, remove the stylet (Figure 4c) and proceed to flush with 5 mL to 10 mL of normal saline solution for adults and 2 mL to 5 mL for children (Figure 4d) and connect the infusion set (Figure 4e). Secure and stabilise the needle to forestall inadvertent dislodgement or bending of the intraosseous needle (Figure 4f).

Some of the strategies used to confirm proper placement of the intraosseous needle include checking for the stability of the needle in the bone, aspiration of marrow, ability to flush with a normal saline solution without extravasation and good intravenous flow rate. It should be noted that some resistance to flushing is expected because unlike a vein, the bone marrow is not distensible. Inability to aspirate does not always indicate poor placement. Should this occur, continue with a normal saline flush and attempt aspiration again. Colour doppler ultrasound, if available, can be used to confirm flow in the intraosseous space and can detect any extraosseous flow in cases of incorrectly placed intraosseous line.^13^ Lastly, it is important to document the date and time of placement of the intraosseous needle for use to estimate how long the needle has been *in situ*.

## Post-insertion management

The intraosseous device should be removed immediately after gaining adequate intravenous access and should not be kept beyond 24 h. It is therefore essential to monitor the time elapsed since placement. Examine the insertion site frequently for signs of extravasation (subcutaneous oedema, increasing limb size) and change in colour. Monitor the position, fixation and patency of the needle.^13^ It is recommended to do a post-insertion X-ray to assess for possible fractures in children.^13^ To remove the intraosseous line, attach a Luer-lock syringe to the hub and keep the hub and syringe in alignment. Rotate the needle clockwise while pulling it straight out.^12,13^ Immediately once the needle is out of the site, apply sterile dressing.

## Complications

Some of the complications that can occur from intraosseous needle insertion include the following:

- Incomplete penetration of the needle into the medullary space manifested as inability to flush with normal saline solution. This problem could be overcome by gently drilling deeper through the bone cortex.
- Application of excessive pressure on the needle during insertion, forcing it entirely through the bone and out the other side. This risk could be minimised by using appropriate landmarks and keeping the needle perpendicular to the long axis of the bone.
- Extravasation of fluid with the potential to cause compartment syndrome. This can happen by through-and-through penetration of both anterior and posterior cortices of the bone.
- Epiphyseal plate necrosis caused by insertion into the epiphyseal plate in children.
- Infection manifested as cellulitis, abscess and osteomyelitis.
- Fat embolism from mobilisation of fat from the bone marrow. Air embolism from air injection along with medication or failure to properly flush line.
- Bent intraosseous needle which may become difficult to remove and require surgical removal.
- Long bone fracture.
- Vascular injury of the extremity.
- Penetration of the mediastinal structures or space in cases of sternal puncture, which can cause pneumothorax, right ventricular laceration with resultant hemopericardium and cardiac tamponade.

## Intraosseous medications

**FIGURE 5:**
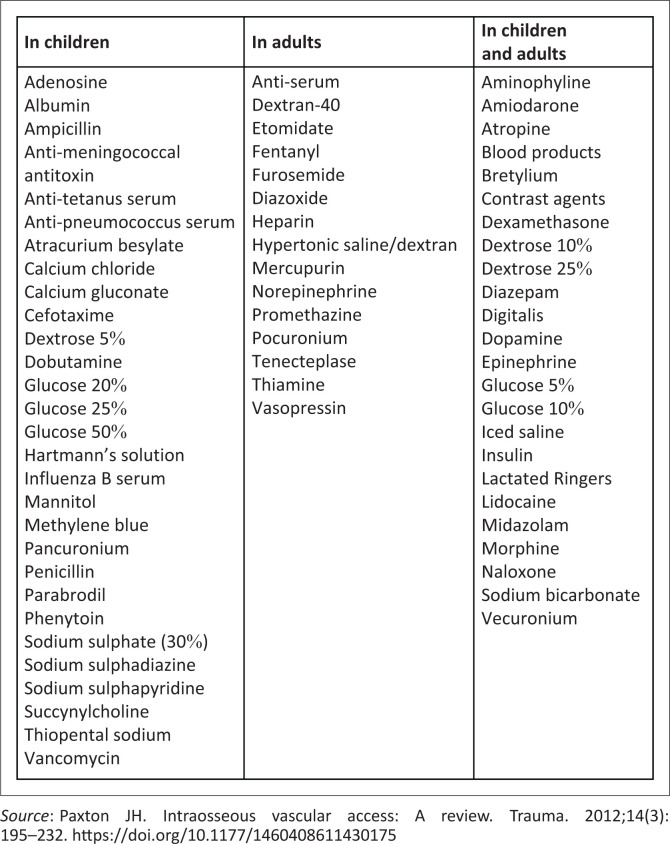
Medications that are safe for intraosseous administration.

Most drugs administered intravenously have equivalent absorption and bioavailability when given through the intraosseous route.^8,14^ With some drugs, however, there is a ‘depot effect’ with the drug lingering in the medullary space that results in a longer time to reach peak concentration and lower peak concentration. The depot effect can be minimised with a 10 mL normal saline flush of the intraosseous needle immediately after medication administration, and higher doses of such drugs may be needed for intraosseous administration.^8^ Medications that may require higher dosage with intraosseous administration include epinephrine, ceftriaxone, amikacin, tobramycin, vancomycin and phenytoin. Refer to Figure 5^8^ for medication that can be used safely in the intra osseous line.

Intraosseous infusion flow rates vary widely with the same needle size and design at the same insertion site.^8^ Some of the factors that influence intraosseous infusion flow rates include infusion pressure, fluid viscosity, radius and length of the needle, hydraulic resistance within the bone marrow and venous system, size of intraosseous space, the presence of obstructive clots and local vascular tone within the medullary cavity and surrounding tissues.^8^ Following administration of medications, the IO cannula must be flushed before and after each medication.

## Conclusion

Intraosseous line insertion is an essential skill in emergencies. Primary health care physicians should be able to perform this procedure as it is lifesaving and usually they are the first contact physicians and 23 other higher-level care are often located long distances away.
